# A computerized Infusion Pump for control of tissue tracer concentration during Positron Emission Tomography *in vivo *Pharmacokinetic/Pharmacodynamic measurements

**DOI:** 10.1186/1756-6649-8-2

**Published:** 2008-05-30

**Authors:** Olof Eriksson, Andreas Wallberg, Stina Syvänen, Raymond Josephsson, Bengt Långström, Mats Bergström

**Affiliations:** 1 Uppsala Imanet, Uppsala, Sweden; 2 Department of Radiology, Oncology and Clinical Immunology, Division of Radiology, Uppsala University, Sweden; 3 Department of Pharmaceutical Biosciences, Faculty of Pharmacy, Uppsala University, Sweden; 4 Department of Medical sciences, Clinical Virology, Uppsala University, Sweden; 5 Departments of Biochemistry and Organic Chemistry, Uppsala University, Sweden; 6 Uppsala Applied Science Lab, GE healthcare, Uppsala, Sweden

## Abstract

**Background:**

A computer controlled infusion pump (UIPump) for regulation of target tissue concentration of radioactive compounds was developed for use in biological research and tracer development for PET.

**Methods:**

Based on observed tissue or plasma kinetics after a bolus injection of the tracer an algorithm calculates the infusion needed to obtain a specified target kinetic curve. A computer feeds this infusion scheme into an infusion pump connected to an animal via a venous catheter. The concept was validated using [^11^C]Flumazenil administrated to Sprague-Dawley rats where the whole brain distribution and kinetic of the tracer was measured over time using a microPET-scanner. The accuracy and precision of the system was assessed by producing steady-state levels of the tracer and by mimicking kinetics after oral administration.

**Results:**

Various kinetic profiles could be generated, including rapid achievement of constant levels, or step-wise increased levels. The resulting tissue curves had low deviation from the target curves according to the specified criteria: AUC (%): 4.2 ± 2.8, Maximal deviation (%): 13.6 ± 5.0 and R^2^: 0.95 ± 0.02.

**Conclusion:**

The UIPump-system is suitable for use in PET-research for assessment of PK/PD properties by simulation of different tracer tissue kinetics in vivo.

## Background

Functional imaging for the assessment of the *in vivo *pharmacokinetics and pharmacodynamics (PK/PD) of drugs either preclinically or in early clinical trials has become an important step in pharmacological research and drug development. Positron Emission Tomography (PET) is a noninvasive imaging tool that allows the assessment of PK/PD parameters directly at the level of the drug target, as opposed to traditional models where tissue PK/PD is estimated indirectly via knowledge of plasma concentrations and/or through measurements of biomarkers as indicators of target modulation. With the administration of radiolabeled drugs, PET allows the direct measurement of tissue concentration of radioactivity, and through knowledge of the specific radioactivity, ratio of radioactivity concentration and concentration of non-radioactive compound, the drug concentration kinetic profile can be obtained with high accuracy. Alternatively, with the administration of a PET tracer with high affinity and specificity for the target, parameters can be extracted from the tissue kinetic profiles which are relevant for target expression, notably binding potential which is proportional to B_max_/k_D_.

During a tissue PK PET scan the labeled drug is commonly administered to the subject by an i.v. bolus injection. After the absorption phase, the tracer level in tissue reaches a maximum and usually starts to decrease due to metabolism and/or washout. Eventually, the tracer is eliminated through the main excretion routes, the liver or the kidney and the urinary bladder. However, most drugs are administered orally which generates an entirely different tissue kinetics as compared to i.v. By recording the kinetics of radioactivity in plasma during the PET scan, the exchange parameters between plasma and tissue can be estimated and applied on the plasma PK from the oral drug administration and thereby calculate the tissue PK of the cold drug [1]. Alternatively it should be possible to generate an i.v. administration scheme which simulates the PK profile obtained from oral administration and thereby direct observe the proper tissue concentration profile in the PET study.

A concept has been suggested in which the radiotracer is administered such to obtain a steady state in tissue and hence constitute the baseline, from which perturbations due to for example a blocking compound, can be identified and consequently quantified.

Various methods have been used to approach the issue of controlling blood concentrations of drugs or tracers. Usually, these techniques have aimed to achieve a steady state concentration of the radiotracer/compound in the blood/plasma. The most common approach is the bolus injection followed by a constant infusion [2-4]. This method is simple but has some drawbacks; it often takes some time until steady-state is achieved, with over- or under-exposures at early time points, and may yield large deviations from targeted concentrations after long time periods. This approach works well if you are only interested in achieving a steady state of a radiotracer in the plasma or in some target tissue. It can not be used to produce more complex TACs. This method also usually needs more time to reach the steady state, at least if the bolus curve is decaying exponentially which usually is the case. Constant subsequent infusion can not compensate well for the washout in this case. If the bolus curve would decay linearly however, then constant infusion would compensate perfectly.

Target controlled infusion (TCI)-systems usually aim to set the tissue concentration of a drug to a steady state level by administrating a bolus followed by exponentially declining infusion rates, where the subsequent infusion parameters are determined from a model of the PK/PD parameters of the compound in the subject [5,6]. This method has been sparsely used in combination with PET [7].

In this study we present a novel approach for controlling tissue concentration of either drug or PET tracer in a PET study. The method is versatile and can be applied with a variety of tracers in different tissues and allows an arbitrary tissue kinetic curve to be generated. The computerized infusion system, UIpump, utilizes an algorithm that compensates for the difference between the bolus curve and the finally desired curve at each discrete time point. The method is designed to infuse a short bolus injection followed by subsequent discrete infusions. The subsequent discrete infusions are designed to compensate the elimination of the tracer by modifying the concentration to the level of the desired curve. The tissue level of radioactivity and mass concentration can be determined at will and hence the total administered amount (μg/kg) over an entire study can be ensured to be below toxicity levels respectively such as the tracer dose by itself is not perturbing the target system [8]. The concept was validated using a well known radiotracer, [*N*-methyl-^11^C]flumazenil which binds to central benzodiazepine receptors (BZR) with high affinity and specificity.

## Methods

### Radiochemistry

[*N*-methyl-^11^C]flumazenil ([^11^C]-ROB) is a selective central BZR antagonist [9] and the most extensively used BZR tracer for PET. [^11^C]ROB was synthesized as described previously [10]. The radiochemical purity of each batch was greater than 95%. The specific radioactivity was typically 52 ± 20 MBq/nmol.

### Animals

Adult male Sprague-Dawley rats weighing 450–600 g were housed under standard laboratory conditions (20°C and 50% humidity) and maintained under a 12-h light-dark cycle with free access to food and water. The animal studies were approved by the local Research Animal Ethics Committee (permission C 153/4). Animals were initially anesthetized using 3.2% isoflurane and continuous anesthesia was maintained by 2.4% isoflurane (600 ml/h air) through a face mask. Temperature was measured and breathing rate was monitored visually. Radiotracers were administered through an i.v. catheter in the tail-vein.

### Infusion pump system

The UIpump system consists of a programmable Univentor 864 Infusion Pump (AgnThos, Sweden) connected to a personal computer. The infusion pump is controlled by UIPump, a custom made Visual Basics-based program on the PC.

Calculation of the UIPump input function is performed through the following equations:

*C*_*target *_(*t*) = *H*_*TR*_(*t*) ⊗ *C*_*infusion *_(*t*)

where C_infusion _defines the infusion rate from the pump, C_target _the tissue kinetics and H_TR _the pump function. The pump function (impulse response) is a reflection of how the body is handling an infinitesimally short exposure, but also includes aspects of the pump and tubing.

If C_infusion _is a short intravenous injection (<5 seconds) it can be approximated to be a Dirac impulse, δ(t), which allows the pump function to be derived from the tissue kinetic curve obtained from this bolus injection.

*C*_*target*-*bolus *_(*t*) = *H*_*TR*_(*t*) ⊗ *δ*(*t*) = *H*_*TR *_(*t*)

where ⊗ is a convolution operator. The intravenous input C_infusion _needed to achieve an arbitrarily chosen C_target _can now be determined from

*C*_*target *_(*t*) = *H*_*TR*_(*t*) ⊗ *C*_*infusion *_(*t*)

where H_TR _is known from the bolus-experiment and C_target _is a designed target tissue concentration curve. H_TR _and C_target _are discretized by resampling the functions at 1 second intervals. Shorter intervals improve the accuracy but also increase the calculation time and amount of data. C_infusion _is found in discrete form (also in 1 second intervals) by deconvolution with the condition to minimize

*NORM*(*H*_*TR*_(*t*) ⊗ *C*_*infusion *_(*t*) – *C*_*target *_(*t*)), where *C*_*infusion *_(*t*) ≥ 0

The deconvolution is performed by using the algorithm *lsqnonneg *(MatLab, MathWorks)) which solves the least square problem with the constraint that all terms are positive or equal to zero. The solution describes the infusion scheme in a discrete form with time intervals of 1 sec.

### MicroPET studies

The imaging studies were performed using a calibrated microPET-R4 scanner (Concorde Microsystems, Knoxville, TN). The scanner provides a 10 × 8-cm field of view (FOV), and the scanner is capable of an axial and trans-axial resolution of 2 mm. A spiraling ^68^Ge point-source was used to acquire 20 minute transmission scans. 3D Emission scan data were acquired in list mode without pre-specified dynamic framing. The images were reconstructed by using 2D filtered back projection (Hanning filter 4.0 mm). Dead time correction and random correction were performed for all scans.

The images were analyzed using ASIPro version 3.2 (Concorde Microsystems Inc., Knoxville, TN). For each emission scan regions of interest (ROIs) were drawn over the whole brain on five different coronal slices and combined to form a volume of interest (VOI). The resulting time-activity curves (TACTs) were normalized by dividing each data point with the value of the point with the maximal activity (to allow for inter-scan comparison).

#### Bolus Curve Acquisition

20–30 MBq/kg were administered as an i.v. bolus injection. The target tissue was monitored by PET for 60 minutes.

#### Infusion Curve Acquisition

In total 9 animals received an integrated dose of 400–600 MBq/kg over 120 minutes. The effective dose (when radioactive decay is accounted for) is much smaller, with the exact biological dose depending on the infusion scheme.

### Data Analysis

The difference between the targeted curve and the resulting curve is one measure of the functionality and accuracy of the infusion system. Three criteria were used to determine the discrepancies between the experimental and target curves:

#### AUC_diff_

The area under curve (AUC) is an important concept in pharmacology and reflects the total dose exposure in the tissue over time. In this case AUC_diff _(%) equals (AUC_exp_-AUC_target_)/AUC_target _*100 and is a measure of the percent difference of the total uptake dose.

#### DEV_max_

The data point in the experimental data set with the largest deviation compared to the target curve (in %) is defined as the maximal deviation DEV_max_. DEV_max _is calculated from dividing the largest deviation by the value of the target curve at the current time point.

#### R^2^

The residual sum of squares between the experimental and target curve was calculated as an additional control of accuracy and precision.

## Results

### Bolus Curve Acquisition

**Figure 1:**
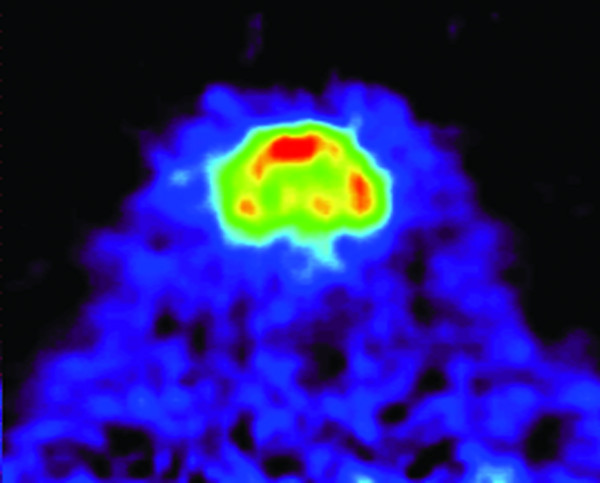
Coronal slice (summed over 45 minutes) showing brain uptake of [^11^C]ROB (injected amount 24 MBq/kg) in Sprage-Dawley rat (anesthetized with 2.4% isoflurane).

Uptake of [^11^C]ROB after i.v. injection was high in the whole brain, combined with low uptake in tissues bordering to the brain. Highest activity levels could be seen in the frontal cortex, thalamus and hippocampus, consistent with the known distribution of the target molecule complex, GABA-receptor (figure 1).

**Figure 2:**
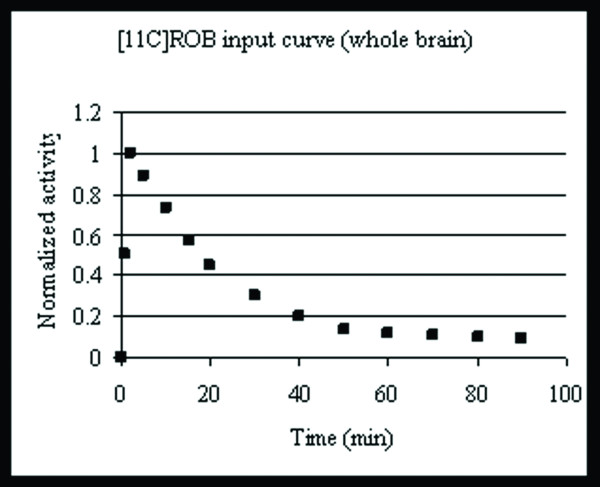
**Average TACT-curve after instantaneous bolus injection of [^11^C]ROB in several animals.** The curve is measured over whole brain. The modeled curve is used as the input function for the calculation of the infusion schemes.

Bolus curves (not shown here) from i.v. injections of [^11^C]ROB in four different SPD rats were averaged and smoothed to generate an averaged bolus curve to be used with the UIPump system (figure 2). The resulting brain curves showed a peak within a few minutes, followed by monoexponential elimination with a halflife of 17.3 min.

### Infusion Curve Acquisition

#### Steady state of radiotracer in whole brain

**Figure 3:**
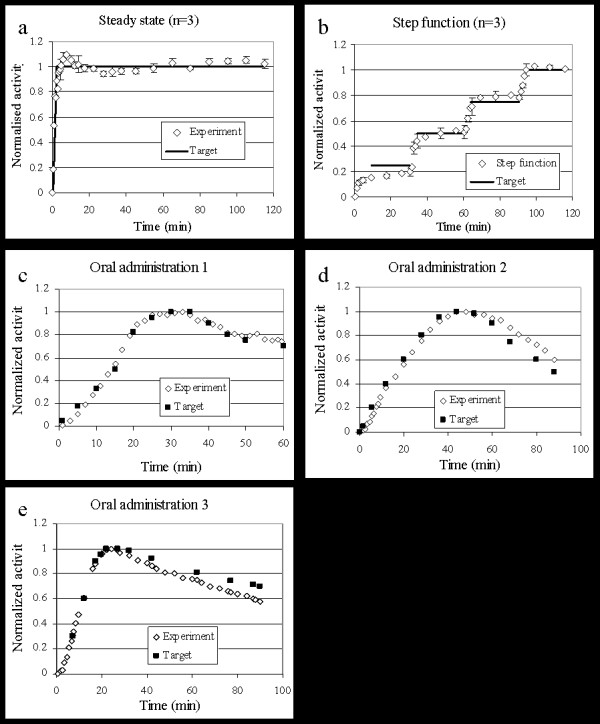
a) Steady state curves (n = 3), b) Step function (4 steady state levels, n = 3), c-e) Simulating uptake after oral administration.

The pump was set to ensure a constant tracer concentration in tissue for up to 120 minutes. The average of 3 experiments is demonstrated in figure 3a. Within 5 minutes, a value within 10% of the steady level had been achieved, and remained close to the plateau for the remaining time.

#### Generating Step Function curve in whole brain

Step function curves (n = 3) were generated using a target curve which consisted of four increasing steady state levels. The levels, 0.25, 0.5, 0.75 and 1.0 (in normalized activity), were each 30 minutes long. The pump could generate a nice stepwise increase of tracer in the brain, although not with a prompt change between the levels.

#### Mimicking Oral Administration uptake in whole brain

Three different curves simulating delayed target tissue uptake (compared to i.v. administration) were selected to investigate the versatility of the infusion system. All three experimental TACTs show the distribution of [^11^C]ROB in whole brain over time which was very close to that desired.

The implementation of UIPump for these target curves using [^11^C]ROB resulted in the whole brain VOI TACTs in figure 3a–e. The functionality and accuracy criteria for the different experiment are presented in table 1. In general the agreement was good between aimed and achieved brain concentration profiles. The AUC-difference was in most cases below 5% and the maximum deviation below 10% except in two cases where the deviations at late time points were 20 and 17%.

**Table 1:** Difference between experimental curve and target curve for all performed studies according to three pre-specified criteria.

**Target curve**	**AUC_diff_(%)**	MAX_dev_(%)*	**R^2^**	**Comment**
Steady state (n = 3)	1.0	9.6	0.92	Figure 3a
Step function (n = 3)	6.3	12.2	0.96	Figure 3b
Oral adm. 1	4.2	8.7	0.98	Figure 3c
Oral adm. 2	1.7	20.3	0.95	Figure 3d
Oral adm. 3	7.6	17.2	0.96	Figure 3e
Average ± stdev	4.2 ± 2.8	13.6 ± 5.0	0.95 ± 0.02	

*At very low concentrations (beginning of scans) even small actual errors gives very large percentual errors. The MAX_dev _is therefore calculated from data points >15 minutes into the scan.

## Discussion

The development of a computerized pharmacokinetic model-driven infusion device was initially investigated in the late seventies and early eighties [11,12]. It was shown that it was possible to attain the desired plasma concentration of an intravenously administered drug by using a computer-controlled pump programmed with the known pharmacokinetic properties of the drug. The UIpump bolus curve effectively replaces the need for detailed PK parameters, making it suitable for new and relatively uncharacterized tracers. However, a prerequisite is that the tissue kinetic profile is first determined with a bolus injection as a means to set the pump response function. [^11^C]ROB was used as a model tracer due to its well described PK/PD (1 tissue-compartment) and its availability. The system has been used with other tracers with more complicated PK/PD (described by 2 tissue-compartments) with promising results. However, there will be a limit for the complexity the system can handle. The system has not yet been tested with tracers with high non-specific binding or tracers whose PK/PD is described with more than two tissue compartments.

The plasma concentration of intravenous drugs or tracers after a bolus peaks virtually instantaneously; however, the peak brain concentration of the drug occurs later when the brain concentration equilibrates with the central plasma compartment. This delay is due to various biological processes (passing the blood-brain barrier etc.). For many studies it is therefore inadequate to simply control the plasma concentration. The UIpump system can control either the concentration in the plasma or the concentration at the site of drug effect, depending on the study design. Hence this system could optimize rather complicated schemes with rapid switches between different levels of drug in the brain.

Sometimes it may be valuable to simulate other uptake curves. PET tracers are very seldom given orally (due to the high concentration of radioactivity in the gastro-intestinal tract and therefore a high local radiation dose). Uptake curves simulating oral administration can be performed with target controlled infusion to better simulate drugs PK in target tissues e.g. for perturbation or blocking studies.

### Blocking studies

When characterizing a novel pharmacological drug with PET, sometimes a blocking study is performed. A target specific tracer is administered before and after the patient is treated with the drug. Difference in uptake compared to a control then reveals pharmacological parameters for the drug, but only at equilibrium in the tissue. By target controlled infusion the tracer levels can be held at a steady state while a drug is administered. Perturbations of the steady state resulting from interactions of the drug at pharmacological levels can be followed over time, from the initial effect until equilibrium. This concept has been used with our pump system to allow careful evaluation of how drugs are affecting the PgP-system at the blood-brain-barrier [13,14]. Hence the pump has ensured a constant brain level of a tracer which is a substrate for PgP, and therefore has limited access to the brain. At a predefined time a challenge has been made with a pharmacological dose with a PgP-inhibitor, allowing the tracer increase to reveal time course and magnitude of inhibition of the PgP.

### Constant levels of therapeutic doses – anesthetic agents

The use of rate-controlled delivery systems in anesthesia has long been exploited [15,16]. Most anesthetic compounds have a critical dose range. A large dose can harm the patient, while a low dose may be insufficient for proper anesthetic effect. Instead of monitoring the plasma levels it can be desirable to control the concentration of the agent in the tissue in which it exerts its effect. This is possible when using an anesthetic agent at pharmacological levels in combination with its radioactively labeled version in trace amounts. An example of this application of UIpump was described earlier [17].

### Biological variation

The biological variation between individuals sets the limit for the accuracy of the UIpump. Comparisons have shown the difference between bolus curves to be around 10% for Sprague-Dawley rats (data not shown here). Due to the long anesthesia, the animals in the study are used only for one tracer administration, preventing the bolus curve from being obtained from the same animal as the infusion curve. For studies in higher species or clinical settings the bolus curve and the infusion regimen can be performed in the same individual, which resolves this issue.

## Conclusion

The UIPump system is currently in use with different tracers in several animal models for preclinical PET studies. It is a functional tool for applications in PET PK/PD experimental setups.

## Competing interests

The authors declare that they have no competing interests.

## Authors' contributions

OE carried out the programming, the microPET experiments, the data analysis and drafted the manuscript. AW carried out the programming and the microPET experiments. SS participated in the data analysis. RJ participated in the design of the study. BL conceived of the study, and participated in its design and coordination and helped to draft the manuscript. MB conceived of the study, and participated in its design and coordination and helped to draft the manuscript. All authors read and approved the final manuscript.

## Pre-publication history

The pre-publication history for this paper can be accessed here:


